# The LH:FSH Ratio in Functional Hypothalamic Amenorrhea: An Observational Study

**DOI:** 10.3390/jcm13051201

**Published:** 2024-02-20

**Authors:** Magdalena Boegl, Didier Dewailly, Rodrig Marculescu, Johanna Steininger, Johannes Ott, Marlene Hager

**Affiliations:** 1 Clinical Division of Gynecological Endocrinology and Reproductive Medicine, Department of Obstetrics and Gynecology, Medical University of Vienna, 1090 Vienna, Austria; magdalena.boegl@meduniwien.ac.at (M.B.); johanna.steininger@meduniwien.ac.at (J.S.); marlene.hager@meduniwien.ac.at (M.H.); 2 Faculty of Medicine Henri Warembourg, University of Lille, CEDEX, 59045 Lille, France; didier.dewailly@orange.fr; 3 Department of Laboratory Medicine, Medical University of Vienna, 1090 Vienna, Austria; rodrig.marculescu@meduniwien.ac.at

**Keywords:** hypogonadotropic hypogonadism, functional hypothalamic amenorrhea, gonadotropin-releasing hormone, polycystic ovary syndrome, LH:FSH ratio

## Abstract

Background: In functional hypothalamic amenorrhea (FHA), luteinizing hormone and follicle-stimulating hormone levels show high interindividual variability, which significantly limits their diagnostic value in differentiating FHA from polycystic ovary syndrome (PCOS). Our aim was to profile the LH:FSH ratio in a large sample of patients with well-defined FHA. Methods: This observational study included all consecutive patients with FHA presenting to the Department of Gynecologic Endocrinology and Reproductive Medicine, Medical University of Vienna, between January 2017 and August 2023. The main parameters of interest were the LH level, the FSH level, and the LH:FSH ratio. In a subgroup analysis, we compared the LH:FSH ratio of patients with PCO morphology (PCOM) on ultrasound with that of patients without PCOM. Results: A total of 135 patients were included. Only a minority of patients revealed FSH and LH levels ≤ 2.0 mIU/mL (13% and 39%, respectively). Most patients (81.5%) had an LH:FSH ratio ≤ 1.0, while a minority (2.2%) had a ratio ≥ 2.1. The LH:FSH ratio was similar in patients with and without PCOM. Conclusion: In a well-defined FHA sample, the LH:FSH ratio was ≤ 1 in most patients. The LH:FSH ratio may prove useful in distinguishing FHA from PCOS but needs further investigation.

## 1. Introduction

Secondary amenorrhea is defined as the cessation of previously regular menstruation for a period of more than three months or previously irregular menstruation longer than six months [1] and affects about 4% of women in the general population [2]. Functional hypothalamic amenorrhea (FHA) and polycystic ovary syndrome (PCOS) are two of the most common underlying conditions [3]. FHA is commonly associated with stress [4], vigorous exercise, weight loss, and psychological disorders [5], leading to suppression of the hypothalamic–pituitary–ovarian (HPO) axis [6], which in turn disrupts follicular growth and ovulation. The resulting hypoestrogenism has profound effects on cardiovascular health [7], bone density, and fatigue and decreases libido [8]. In many cases, the onset is attributed to the interplay of various etiologies, which are potentially influenced by genetic or epigenetic predispositions [9]. However, correcting or ameliorating the stressors can fully restore ovulatory ovarian function [10]. 

PCOS is an important and sometimes difficult differential diagnosis [11]. PCOS is diagnosed using the Rotterdam criteria as recommended in the “International evidence-based guideline for the assessment and management of polycystic ovary syndrome 2018” [12]. The Rotterdam criteria require the presence of two of the following features: oligo-anovulation, signs of hyperandrogenism, and polycystic ovaries (≥ 12 follicles measuring 2–9 mm in diameter and/or an ovarian volume >10 mL in at least one ovary) visible on ultrasound [12,13]. However, according to the recently published “International Evidence-based Guideline for the assessment and management of Polycsytic Ovary Syndrome (PCOS) 2023”, anti-Muellerian hormone (AMH) levels in plasma can be determined instead of sonographic measurement of the follicular cysts [14]. Anti-Muellerian hormone is commonly used to assess the ovarian follicular reserve and to identify PCOM in adults.

Since up to 50% of women with FHA reveal polycystic ovarian morphology on ultrasound [4,15], which is also accompanied by increased AMH levels, these patients can easily be misdiagnosed as PCOS [16]. To date, four different PCOS phenotypes have been identified: Phenotypes A, B, C, and D. Non-hyperandrogenic phenotype D (PCOS-D) requires only anovulation and PCO morphology and remains the most difficult to distinguish from FHA with PCOM [15]. Recent data show that with an AMH threshold of 3.2 ng/mL, 34.8% are classified as phenotype D [17], The similarities such as secondary amenorrhea, PCO morphology on ultrasound/increased AMH levels, and infertility make it very difficult to differentiate between the two conditions. The fact that there are no highly reliable parameters for the differential diagnosis between FHA and PCOS has been underlined by a recent review [11].

In FHA, the imminent cause of amenorrhea is a disrupted frequency pattern of gonadotropin-releasing hormone (GnRH) secretion [18]. Exposure to stress activates the hypothalamic–pituitary–adrenal axis, leading to an elevated secretion of corticotropin-releasing hormone (CRH) and glucocorticoids such as cortisol [19], which inhibit GnRH secretion and release. As a result, LH (luteinizing hormone) and FSH (follicle-stimulating hormone) levels decrease and then are no longer sufficient to maintain folliculogenesis and ovulatory ovarian function [10]. Thus, it seems reasonable to use LH and FSH as parameters to diagnose FHA and differentiate between FHA and PCOS. According to several studies, this seems feasible for LH [11,16] but controversies exist for the use of FSH [11,16]. Generally, while the LH profile has been extensively studied in FHA women, less is known about the role of FSH in women with FHA [20,21]. The pattern of GnRH secretion appears to be an important factor in regulating gonadotropin subunit gene expression, gonadotropin synthesis, and hormone secretion [22]. It is thought that in hypothalamic–pituitary–ovarian axis dysfunction, an inadequate production of GnRH by the hypothalamus (i.e., slow frequency of GnRH pulses) leads to a decreased secretion of LH and, to a lesser extent, of FSH, since reduced GnRH pulsatility favors FSH secretion [23]. Consequently, the LH:FSH ratio would theoretically be lower in FHA than in other situations and could be used as a diagnostic criterion. We have indeed previously reported that a threshold of 0.96 has a very high specificity to discriminate between women with FHA and PCOM and women with phenotype D of PCOS [16].

However, since FSH levels in FHA patients vary from one study to the other and since no one has previously primarily focused on the LH:FSH ratio, we aimed to investigate this parameter in our large patient population with well-defined FHA. Our goal was to define its distribution and to search for relationships with various hormones in women with FHA to shed some new light on the pathophysiologic aspects of FHA. 

## 2. Materials and Methods

Study design: we conducted a single-center, retrospective observational study to investigate the LH:FSH ratio in patients with well-defined FHA. 

Study population: This observational study included 135 consecutive patients with FHA presenting to the Clinical Department of Gynecological Endocrinology and Reproductive Medicine, Medical University of Vienna, Austria, from January 2017 to August 2023. The FHA definition includes the presence of secondary amenorrhea for at least six consecutive months and a negative progestogen challenge test. Women with pregnancy, hypothyroidism, acne and hirsutism, hyperprolactinemia, and other organ-related pituitary dysfunctions (by MRI) were excluded from study participation. 

Reasons causing amenorrhea were extensively described in previous studies [4,21,24,25,26]. In detail, all women presenting with FHA had experienced reasonably regular menstrual cycles prior to the manifestation of amenorrhea. A weight loss exceeding 10 kg prior to the onset of amenorrhea was considered significant. Furthermore, a body mass index (BMI) below 18.5 kg per square meter, as per the established criteria for classifying underweight individuals, indicated a likelihood of FHA due to underweight status. Diagnoses of eating disorders were made in accordance with the ICD-10 criteria. Each participant classified as an “exerciser” when engaged in physical activity for a minimum of 10 h per week, which encompassed various forms of exercise including dancing, aerobics, biking, and more, or running at least 30 miles per week. It is imperative to acknowledge the presence of emotionally distressing events leading to the onset of amenorrhea, including familial, scholastic, occupational, or psychosocial stressors (psychiatric disorders were ruled out using DSM IV criteria). None of the women displayed clinical manifestations of hirsutism or acne.

Parameters analyzed: The AKIM software (SAP-based patient management system at the Medical University of Vienna) was used for data acquisition. In addition, the following serum parameters were analyzed: anti-Müllerian hormone (AMH), total testosterone, sex hormone-binding globulin (SHBG), prolactin, estradiol, and thyroid-stimulating hormone. The data was retrieved from the electronical medical database AKIM (based on SAP ERP Release 2005, V33 (01/2021), Walldorf, Baden Würtenberg, Germany).

Blood samples were collected from a peripheral vein during the early follicular phase (cycle days 2–5) after bleeding induction with oral estradiol (2 mg per day) and dydrogesterone (20 mg/day) for 10 days. Laboratory analyses were performed at the Department of Laboratory Medicine, Medical University of Vienna, in compliance with ISO 15189 quality standards acc (International ISO standard, number 15189, Akkreditierung Austria, Stubenring 1, 1010 Vienna, Austria, 2012) ording to previous publications [4,16,27,28]: estradiol, follicle-stimulating hormone (FSH), luteinizing hormone (LH), anti-Mullerian hormone (AMH) and sex hormone-binding globulin (SHBG) were measured by the corresponding Cobas electrochemiluminescence immunoassays (ECLIAs) on Cobas e 602 analyzers (Roche, Mannheim, Germany). All specific tests used were described previously by Beitl et al. [16].

The baseline patient characteristics collected included age at admission, body mass index (BMI), gravidity, parity, and follicle number per ovary (FNPO), which was determined by ultrasound using an “Aloka Prosound 6” ultrasound machine and an “UST-9124 Intra Cavity transducer” (frequency range 2.0–7.5 MHz; Hitachi, Wiener Neudorf, Austria). The threshold for defining follicular excess was set at 12 follicles per ovary, as recommended for an ultrasound machine with probe frequency range <8 MHz [29].

Statistical Analysis: We present categorical data as numbers and frequencies, and continuous data as median and interquartile range (IQR). We used the analysis of variance (ANOVA) for between-group comparisons. Univariate correlations between some variables were sought using Spearman’s non-parametric test. To evaluate possibly associated factors with categorical data, univariable binary regression models were used. All significant parameters were then entered into a multivariable binary regression model. For these models, odds ratios (ORs), their 95% confidence intervals (95% CI), and *p*-values are provided. The IBM Statistical Package for Social Science software (SPSS 28.0) was used for all statistical tests. *p*-values < 0.05 were considered significant. 

## 3. Results

A total of 135 consecutive patients with FHA were included in this study. Table 1 shows the baseline characteristics of the study patients. 

**Figure 1:**
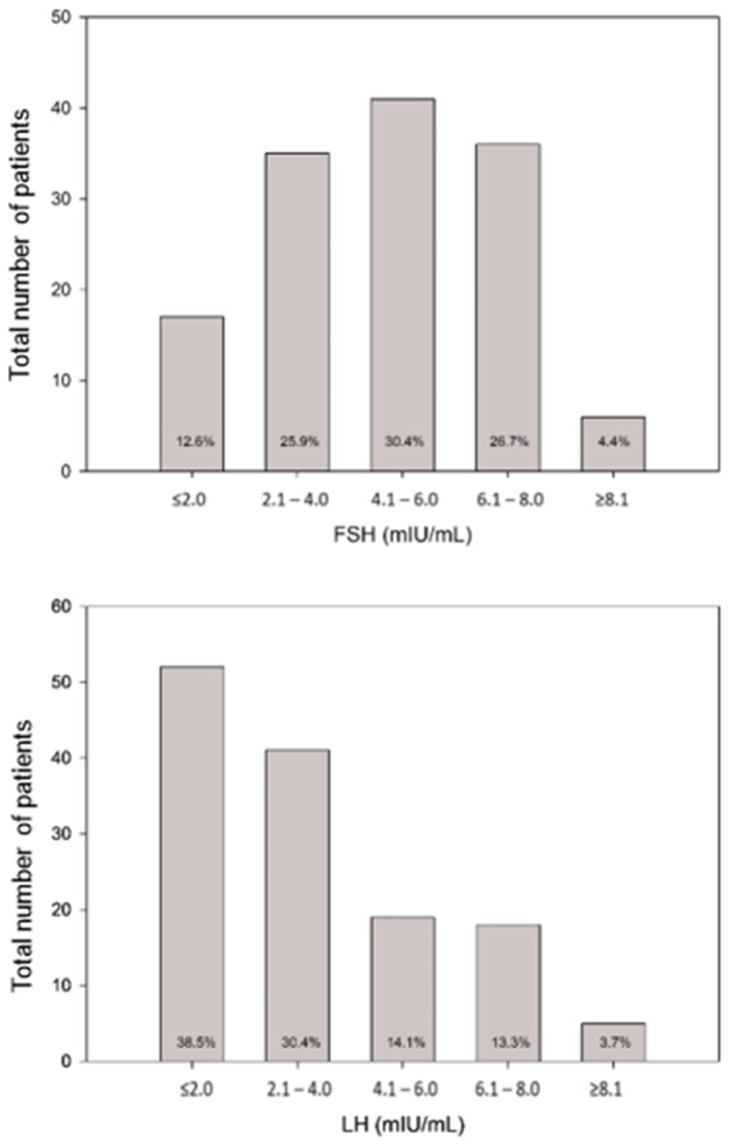
Distribution of FSH and LH levels (mIU/mL) at initial diagnosis of FHA.

**Figure 2:**
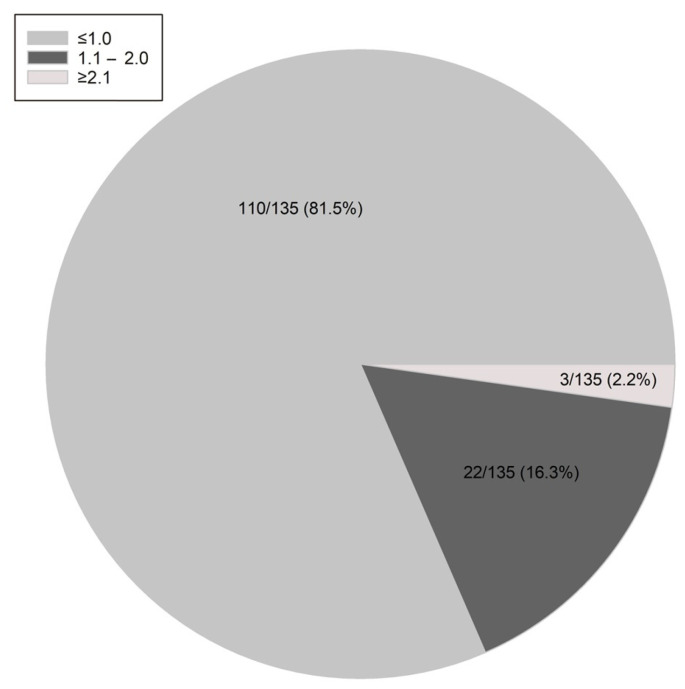
Distribution of the LH:FSH ratio at the initial diagnosis of FHA.

The distribution of FSH and LH values is shown in Figure 1. FSH was ≤ 4.0 mIU/mL and ≤ 2.0 mIU/mL in 38.5% and 12.6%, respectively, whereas this was the case for 68.9% and 38.5% of LH levels, respectively. The LH:FSH ratio was ≤ 1.0 in most patients (81.5%), whereas a value ≥ 2.1 was found in only 2.2% (Figure 2). 

The following significant positive correlations (*p* < 0.05) between serum parameters were found (Table 2): FSH and LH; FSH and estradiol; FSH and AMH; LH and the LH:FSH ratio; LH and estradiol; LH and testosterone; LH and prolactin; the LH:FSH ratio and prolactin; the LH:FSH ratio and estradiol; the LH:FSH ratio and prolactin; estradiol and testosterone; estradiol and prolactin; as well as testosterone and prolactin. 

In order to detect possible confounders, we then conducted a univariable followed using a multivariable binary regression model using the LH:FSH ratio as the dependent variable (≤ 1 versus >1; Table 3). In the univariable models, higher estradiol and higher LH were associated with an LH:FSH ratio >1, whereas this was only the case for LH in the multivariable analysis (OR 0.520, 95%CI: 0.400–0.675; *p* < 0.001).

In a last step, we compared the 77 FHA women without (57.0%) to the 58 FHA women with PCOM (43.0%) (Table 4). The latter revealed higher AMH values (6.3 ng/mL, IQR 4.9–7.6 versus 2.0 ng/mL, IQR 1.1–2.7; *p* < 0.001) as well as lower SHGB values (67.0 nmol/L, IQR 39.7–94.1 versus 79.4 nmol/L, IQR 63.3–104.0; *p =* 0.008). The ranges of the LH:FSH ratio were similar between the two groups.

## 4. Discussion

In this retrospective analysis, about 13% of FHA patients revealed FSH levels <2.0 mIU/mL, whereas decreased LH levels <2 mIU/mL were found in about 39% of patients. Importantly, over 80% of women revealed an LH:FSH ratio ≤ 1. In addition, an LH:FSH ratio >1 was associated with higher LH levels only. Last but not least, FHA women with PCOM did not reveal an altered LH:FSH ratio.

Before discussing these findings in detail, the focus should be on basic patient characteristics. We consider the fact that only women with well-defined FHA were included a study strength. Notably, excessive exercise was the most common cause for FHA (40.7%) followed by stress (32.6%). A low median BMI of 20.3 kg/m^2^ [10] as well as the low median FSH, LH, and testosterone levels (Table 1) seem typical for FHA patients and are comparable to previous studies [10,15,30,31]. Notably, a negative progestogen challenge test and clear causes for FHA were mandatory definition criteria in our study population. However, since PCOS is an important and difficult differential diagnosis and since there is a lack of clear diagnostic criteria [15], we cannot completely rule out that very few PCOS patients might have been included. Nonetheless, we consider this circumstance only a minor study limitation. 

The main finding was that a relevant proportion of our FHA patients revealed normal FSH and LH levels even though this was more often the case for FSH than for LH. From a pathophysiological perspective, this seems reasonable since reduced GnRH pulsatility has been reported to favor FSH secretion [23]. Notably, FSH levels in FHA patients have been reported to vary from study to study, leading to ambiguity in clinical practice [32]. Given the mentioned pathophysiologic considerations, where a decrease in GnRH pulsatility will likely result in substantially decreased LH levels, but only a modest decline in FSH levels, it seems comprehensible that so many FHA women in our study population revealed an LH:FSH ratio ≤ 1.0 (81.5%). It is noteworthy that, to the best of our knowledge, this parameter has not been reported previously in FHA patients [15]. Our data show that women with an LH:FSH ratio >1 revealed higher LH levels in both the univariable and multivariable analysis, whereas the association with higher estradiol levels was only found in the univariable model (Table 3). It seems of particular interest that no other parameter was of influence. Therefore, a lower LH:FSH ratio may be considered as a specific reflection of the GnRH dysregulation of FHA, i.e., greater suppression of LH than FSH, presumably due to slow GnRH pulsatility [23]. Thus, one might consider the LH:FSH ratio better than LH itself, for which we have no consensual threshold [11], since the ratio also integrates FSH. Moreover, based on the fact that it seems to be influenced by GnRH dysregulation alone rather than other factors, the LH:FSH ratio could help clinical decision making in the future, especially concerning the above-mentioned differential diagnosis to PCOS. Thus, comparative studies, especially challenging FHA vs. PCOS in large populations, are needed in the future. Indeed, although the LH:FSH ratio is not a definition criterion for PCOS [13], an elevated LH:FSH ratio is commonly associated with the presence of PCOS. 

Concerning the correlation analyses (Table 2), the positive correlation between the LH:FSH ratio and LH and between the LH:FSH ratio and estradiol seem to confirm the above-mentioned results and considerations. A higher GnRH pulse frequency also leads to a higher LH pulse frequency [33]. Accordingly, a higher LH level could reflect a better overall GnRH pulse generator function and, thus, better ovarian function reflected by higher estradiol levels would be logical. This is somehow also supported by the positive correlation between LH and testosterone, since a relevant amount of androstenedione, the most important precursor of testosterone, is produced by the theca cells in the ovary [34], under the influence of LH [35]. The positive correlation between estradiol and testosterone is in line with these observations. In addition, the LH:FSH ratio revealed a positive correlation with the serum prolactin level. It has been mentioned that the prolactin level could be considered a “sensor” of the hypothalamic–pituitary dysregulation even when it is within the normal range [36]. As shown previously by our study group, eating disorders and excessive exercise tended to lower prolactin levels in FHA women [37]. Therefore, the lower the LH:FSH ratio, the lower the prolactin level. The relevance of the prolactin level, which also affects metabolism, osmoregulation, immune function, behavior, and many more [38,39], needs to be elucidated in women with FHA in the future. However, despite the fact that prolactin levels do not differ between FHA patients and normally cycling controls, it has been mentioned that prolactin levels might be some kind of “sensor” of the hypothalamic activity as mentioned above. However, since increased prolactin levels are usually an exclusion criterion for FHA and since prolactin also exerts metabolic effects, further studies are needed to elucidate the relevance of prolactin FHA women [36]. It seems noteworthy that in the previous analysis, the presence of PCOM in ultrasound was associated with higher prolactin levels [36], which was not the case in the analysis presented herein. 

The lack of data on adrenal androgens must also be considered as a minor limitation. Several factors are associated with insulin resistance in PCOS, including genetic mutations, lipodystrophy [40], and childhood obesity according to the Bogalusa Heart Study’s findings [41]. In addition, it has been shown that anovulatory patients with PCOS have a higher risk of dysglycemia and hyperinsulinemia compared to oligo-amenorrheic or eumenorrheic patients [42]. Whether the inclusion of data about insulin resistance in FHA would be of relevance remains open for discussion. At least it was shown recently that the majority of women with FHA did not reveal abnormal levels according to the “Homeostasis Model Assessment of Insulin Resistance” (ZITAT EINFÜGEN).

It seems reasonable that higher FSH levels were positively correlated with higher estradiol levels (Table 2). Estradiol is synthetized by granulosa cells through the action of aromatase, which is also present in small growing follicles and is FSH-dependent [35]. Moreover, higher FSH levels were associated with higher AMH levels. This phenomenon has been reported previously [27], which lends support to the hypothesis that the relative FSH deficiency, which is typical for FHA, leads to a decrease in the pool of growing follicles and therefore to a decrease in ovarian AMH production [15,27]. This relationship between FSH and AMH had no impact whatsoever on the LH:FSH ratio.

Once again, a high rate of PCOM in women with FHA was found (43.0%), which is in accordance with previous case series [4,15,20,30,43,44,45]. Although stress sensitivity has been suggested as a possible cause for the high PCOM prevalence [4], the exact mechanism remains unknown. FHA women with PCOM revealed higher AMH levels (Table 4), which has been found previously and seems plausible [15,27], as well as lower SHBG levels, which is a new finding in patients with FHA. SHBG production is lower in PCOS women with insulin resistance [46]. Although FHA patients with PCOM revealed higher HOMA index levels for insulin resistance [28], this might not completely explain the difference in SHBG levels. However, the clinical relevance is questionable, since both groups revealed median SHBG levels within the normal range (with PCOM: 67.0 nmol/L, without PCOM: 79.4 nmol/L). However, and this seems of importance, there was no difference in the main outcome parameter LH:FSH ratio between FHA women with and without PCOM (median 0.8 versus 0.7, respectively; *p* = 0.728; Table 4). Although it has been proposed that some FHA women with PCOM initially had simple PCOS before [4,15,27,28] and that they reveal a hyper-responsiveness of LH to a GnRH bolus similar to PCOS patients [27], the data suggest that in both groups, the demise in GnRH pulsatility was comparable. It is still unclear how many FHA patients with PCOM have underlying PCOS. It would be reasonable if this would apply only to a minority of patients. However, the question of why so many women with FHA reveal PCOM remains open. When talking to other experts, some suggest that PCOM would reflect a different state of ovarian stimulation in these women. However, the data presented herein do not support this hypothesis. 

Concerning limitations, the retrospective study design must be taken into account in addition to the above-mentioned difficulties to completely separate FHA from PCOS patients. However, the large sample size with well-defined FHA (negative progestogen challenge test, normal pituitary MRI, clear cause for FHA) might be considered a study strength. 

## 5. Conclusions

Our data show that an LH:FSH ratio ≤ 1 is found in >80% of women with FHA, whereas most of these patients revealed FSH levels >2 mIU/mL. Thus, physicians should not rely on normal FSH levels to rule out FHA. Notably, this decrease in the LH:FSH ratio seems to be relevantly associated with dysfunction of the hypothalamic GnRH pulsatility. The LH:FSH ratio might also be a promising parameter for the differential diagnosis between FHA and PCOS in the future.

## Figures and Tables

**Table 1:** Basic patient characteristics.

Age (years), median (IQR) ^1^	26 (22;29)
BMI (kg/m^2^), median (IQR) ^1^	20.3 (18.6;22.0)
Gravidity: *n* (%) ^2^	
0	134 (99.3)
1	1 (0.7)
Parity: *n* (%) ^2^	
0	134 (99.3)
1	1 (0.7)
Causes for FHA: *n* (%) ^2,3^	
Stress	44 (32.6)
Excessive exercise	55 (40.7)
Anorexia nervosa	30 (22.2)
Acute weight loss	33 (24.4)
Underweight	24 (17.8)
Duration since last menstrual bleeding (months), median (IQR) ^1^	14 (10;24)
Hormones, median (IQR) ^1^	
TSH (IU/mL)	1.57 (1.12;2.03)
Prolactin (ng/mL)	8.9 (6.6;12.9)
FSH (mIU/mL)	4.7 (3.3;6.5)
LH (mIU/mL)	2.6 (1.3;4.7)
Estradiol (pg/mL)	23 (12;31)
Testosterone (ng/mL)	0.20 (0.13;0.29)
DHEAS (µg/mL)	2.03 (1.40;2.73)
SHBG (nmol/L)	73.0 (55.1;101.8)
AMH (ng/mL)	3.1 (1.6;6.2)
Polycystic ovarian morphology on ultrasound, *n* (%) ^2^	58 (43.0)

Note: ^1^ Continuous data are provided as median and interquartile range; ^2^ categorical data are presented as absolute numbers (*n*) and relative frequencies (percent); ^3^ since more than one cause of FHA (e.g., excessive exercise + stress) was identified in some patients, the sum of the cause distribution exceeds the total number of study patients. BMI = body mass index, TSH = thyroid-stimulating hormone, FSH = follicle-stimulating hormone, LH = luteinizing hormone, DHEAS = dehydroepiandrosterone-sulfate, SHBG = sex hormone-binding globulin, AMH = anti-Mullerian hormone.

**Table 2:** Correlation analyses.

		FSH	LH	LH:FSH Ratio	Estradiol	Testosterone	AMH	Prolactin
FSH	r2	-	0.556	−0.141	0.291	0.045	0.221	0.323
	*p*		*<0.001*	0.103	*<0.001*	0.604	*0.010*	0.134
LH	r2	0.556	-	0.633	0.387	0.218	0.060	0.315
	*p*	*<0.001*		*<0.001*	*<0.001*	*0.011*	0.134	*<0.001*
LH:FSH ratio	r2	−0.141	0.633	-	0.271	0.159	−0.076	0.261
	*p*	0.103	*<0.001*		*0.002*	0.066	0.385	*0.002*
Estradiol	r2	0.291	0.387	0.271	-	0.243	0.076	0.182
	*p*	*<0.001*	*<0.001*	*0.002*		*0.005*	0.386	*0.037*
Testosterone	r2	0.045	0.218	0.159	0.243	-	0.126	0.268
	*p*	0.604	*0.011*	0.066	*0.005*		0.134	*0.002*
AMH	r2	0.221	0.060	−0.076	0.076	0.126	-	−0.051
	*p*	*0.010*	0.134	0.385	0.386	0.134		0.555
Prolactin	r2	0.323	0.315	0.261	0.182	0.268	−0.051	-
	*p*	0.134	*<0.001*	*0.002*	*0.037*	*0.002*	0.555	

Correlation coefficients and *p*-values for Spearman correlations are provided; italic numbers indicate statistical significance.

**Table 3:** Parameters associated with an LH:FSH ratio ≤ 1 in women with FHA. Univariable followed by a multivariable binary regression model.

	LH:FSH Ratio ≤ 1 (*n =* 110)	LH:FSH Ratio > 1 (*n =* 25)	OR (95%CI)	*p*	OR (95%CI)	*p*
Age (years) ^1^	26 (22;29)	24 (22;28)	1.047 (0.956;1.147)	0.321	-	-
BMI (kg/m^2^) ^1^	19.9 (18.6;21.7)	21.2 (18.6;22.3)	0.880 (0.740;1.047)	0.149	-	-
Causes for FHA:						
Stress ^2,3^	33 (30.0)	11 (44.0)	0.545 (0.224;1.327)	0.181	-	-
Excessive exercise ^2,3^	45 (40.9)	10 (40.0)	1.038 (0.428;2.518)	0.933	-	-
Anorexia nervosa ^2,3^	22 (20.0)	8 (32.0)	0.531 (0.203;1.389)	0.197	-	-
Acute weight loss ^2,3^	27 (24.5)	6 (24.0)	1.030 (0.373;2.844)	0.954	-	-
Underweight ^2,3^	19 (17.3)	5 (20.0)	0.835 (0.279;2.503)	0.748	-	-
Duration since last bleeding (months) ^1^	14 (12;24)	12 (8;16)	1.039 (0.998;1.082)	0.065	-	-
FSH (mIU/mL) ^1^	4.7 (3.4;6.3)	4.7 (2.6;6.5)	1.056 (0.868;1.284)	0.588	-	-
LH (mIU/mL) ^1^	2.2 (1.2;3.5)	6.3 (4.0;7.7)	0.499 (0.385;0.647)	<0.001	0.520 (0.400;0.675)	<0.001
Prolactin (ng/mL) ^1^	8.1 (6.4;12.4)	12.0 (8.8;14.2)	0.960 (0.894;1.031)	0.260	-	-
Estradiol (pg/mL) ^1^	21 (11;28)	25 (22;39)	0.956 (0.928;0.986)	0.004	0.975 (0.939;1.013)	0.196
Testosterone (ng/mL) ^1^	0.19 (0.13;0.29)	0.25 (0.14;0.33)	0.015 (0.000;1.030)	0.052	-	-
SHBG (nmol/L) ^1^	74.0 (57.4;99.1)	70.0 (48.6;113.0)	0.998 (0.986;1.010)	0.720	-	-
AMH (ng/mL) ^1^	2.8 (1.6;6.1)	4.5 (2.2;6.2)	0.955 (0.863;1.087)	0.486	-	-
PCOM ^2^	42 (38.2)	13 (52.0)	0.570 (0.238;1.366)	0.208	-	-

^1^ Numerical data are provided as median (interquartile range) and ^2^ categorical data as number (frequency); ^3^ multiple mentions possible.

**Table 4:** Comparison of FHA with and without PCOM.

	PCOM (*n* = 58)	Non-PCOM (*n* = 77)	*p*
Age (years)	26 (22;28)	26 (22;30)	0.299
BMI (kg/m^2^)	20.4 (18.8;22.5)	20.0 (18.4;21.4)	0.190
Prolactin (ng/mL)	9.6 (6.6;13.0)	8.8 (6.7;12.8)	0.964
FSH (mIU/mL)	4.8 (3.5;6.5)	4.7 (3.2;6.2)	0.426
LH (mIU/mL)	2.8 (1.5;5.5)	2.6 (1.3;4.6)	0.290
LH:FSH ratio	0.8 (0.3;1.0)	0.7 (0.4;1.0)	0.728
Estradiol (pg/mL)	24 (11;34)	22 (12;29)	0.719
Testosterone (ng/mL)	0.20 (0.14;0.29)	0.19 (0.13;0.29)	0.881
SHBG (nmol/L)	67.0 (39.7;94.1)	79.4 (63.3;104.0)	0.008
AMH (ng/mL)	6.3 (4.9;7.6)	2.0 (1.1;2.7)	<0.001

Data are provided as median (interquartile range). Please find hormones described in Table 1. A *p*-value < 0.05 was considered significant.

## Data Availability

Data will be made available upon reasonable request.
